# Minimally Invasive Antegrade Fixation of Proximal Phalangeal Fractures with Intramedullary Cannulated Compressive Screws

**DOI:** 10.3390/jcm15093289

**Published:** 2026-04-25

**Authors:** Seung Yun Oh, Seokchan Eun

**Affiliations:** 1 Faculty of Medicine, Dentistry and Health Sciences, The University of Melbourne, Parkville, Melbourne, VIC 3010, Australia; seungyuno@student.unimelb.edu.au; 2 Department of Plastic and Reconstructive Surgery, Seoul National University College of Medicine, Seoul National University Bundang Hospital, 82 Gumi-ro, 173 beon-gil, Seongnam 463-707, Republic of Korea

**Keywords:** proximal phalanx fracture, intramedullary headless screw, cannulated compressive screws, intramedullary fixation, antegrade approach

## Abstract

**Background/Objectives**: Proximal phalangeal fractures account for 38% of all phalangeal fractures, with unstable patterns requiring surgical intervention. Various modalities have been explored, including open reduction and internal fixation, percutaneous K-wire fixation, and intramedullary techniques. This study explores the technical nuances, indication, and outcomes of antegrade cannulated compressive screw (CCS) fixation of proximal phalangeal fractures. **Methods**: This retrospective case series involved 18 closed proximal phalangeal fractures in 16 patients who underwent intramedullary headless screw fixation between January 2018 and December 2023. Records were reviewed for demographics, fracture characteristics, and screw type. With the metacarpophalangeal joint flexed at 60–75°, a 1 cm longitudinal incision was made, the extensor tendon split, and a 0.9 mm guidewire advanced anterogradely along the phalangeal axis under fluoroscopy. A 2.2 mm or 3.0 mm SpeedTip CCS was selected based on phalanx size and advanced until fully buried below the cartilage line. Postoperatively, patients were immobilized in a volar intrinsic-plus splint, transitioned to a gutter splint within five to seven days, and commenced on range of motion (ROM) exercises within one week. Primary outcomes included radiographic union, Total Active Motion (TAM), QuickDASH scores, and postoperative complications. **Results**: All fractures were healed within acceptable radiological parameters and with no postoperative complications. Mean TAM was measured to be 216.0° (SD 7.7°, range 200–230°) and mean QuickDASH was 10.1 (SD 2.8, range 5–16). **Conclusions**: Antegrade intramedullary headless screw fixation demonstrates feasibility, short-term safety, and excellent early functional outcomes for carefully selected unstable proximal phalanx fractures, supporting its role as a minimally invasive alternative in appropriately indicated cases.

## 1. Introduction

Phalangeal fractures are among the most common injuries of the upper extremities accounting for approximately 10% of all fractures [1,2]. Within the hand, the proximal phalanx is the second most commonly injured bone accounting for approximately 15% to 20% of all hand fractures and 38% of all phalangeal fractures [3,4]. The primary goal of treatment is to achieve fracture healing in an acceptable anatomical alignment while preserving the functionality of the hand to allow early mobilization [5]. This balance is critical to prevent stiffness of the distal interphalangeal (DIP) and proximal interphalangeal (PIP) joints in order to preserve the gliding motion of surrounding flexor and extensor tendons [4,5].

Management of these fractures depends on their stability. Stable, non-displaced fractures can often be treated via conservative, non-operative, means with aims of providing sufficient stability while minimizing the risks associated with immobilization such as joint stiffness [6]. However, unstable fractures based on fracture pattern and mechanism of injury, patient characteristics, and functional requirement may require surgical intervention [6,7]. Historically, surgical options have included percutaneous Kirschner-wire (K-wire) fixation, open reduction with plates and screws, and internal fixation with K-wires, screws, and microplates [8]. While these methods can be effective, they each have inherent drawbacks. Plate fixation provides absolute stability but requires extensive soft tissue dissection, which can lead to tendon adhesions, stiffness, and the potential need for second surgery for hardware removal [9]. Whereas K-wire fixation, although less invasive, provides less rigid stability, often requires prolonged immobilization, and carries postoperative complication risks such as pin-site infections and extensor tendon tethering [6,8,9].

To address these limitations, intramedullary fixation using cannulated compressive screws (CCS), also known as intramedullary headless screws (IMHS), has emerged as a reliable and minimally invasive alternative [2,10]. This technique was first popularized for scaphoid fractures by Herbert and Fischer, but in recent years have been adapted for phalangeal and metacarpal fractures [11,12,13]. The minimally invasive nature of this technique offers the advantage of providing rigid internal fixation with minimal disturbance to surrounding soft tissues, tendons, and joints, all of which facilitates early postoperative mobilization with minimal pain [2,6,10,13]. However, this technique also has its own unique set of complications. A primary concern is the creation of a defect in the articular cartilage at the screw’s entry point, which could potentially lead to joint damage over time [14,15]. Furthermore, in a case series by Kupperman [16] hardware-related issues such as screw unraveling has been noted, but particularly so in the dense bone of younger patients. Several studies attribute this hardware complication to the self-drilling nature of headless screws resulting in “catching” to the dense cancellous bone in younger patients [17]. This in combination with rotational momentum provided upon insertion of the screw increases its risk to unravel [17,18,19,20].

Despite the growing use of these implants and the existing literature elucidating its effectiveness, technical precision and precaution is highlighted in avoiding adverse outcomes. Furthermore, in a recent systematic review by Dagi [21] states that the existing literature supporting the efficacy and safety of intramedullary screw fixation is limited, requiring further research. As such, this study aims to evaluate the clinical complications and outcomes following antegrade cannulated compressive screw fixation of proximal phalangeal fractures and to also provide suggestions on how to avoid the potential complications associated with this surgical technique.

## 2. Materials and Methods

This study is a single surgeon (S.C. Eun) retrospective case series review conducted on all patients who underwent intramedullary headless screw fixation for proximal phalangeal fractures between January 2018 to December 2023. This study received approval from the Seoul National University institutional review. All patients who were treated with intramedullary headless screw fixation during this period were included. Cases with complex fractures such as comminuted and long oblique fractures were considered for alternative techniques. There was one case involving two short oblique fractures in the second and third digits. This case was accepted given the fracture angles remain biomechanically amenable to intramedullary compression fixation, consistent with the existing literature for this technique [22,23]. Patient medical records, operative reports, and radiographs were reviewed to collect data on demographics, fracture characteristics, and screw type. Informed consent was obtained from all patients prior to their procedure. The primary outcomes assessed in this study were radiographic union, functional recovery, patient-reported outcomes, and postoperative complications. All analyses were conducted on IBM SPSS Statistics (ver 30.0).

### 2.1. Surgical Technique

All procedures were performed following sterile preparation of the operative extremity and manual reduction in the fracture under fluoroscopic guidance. With the metacarpophalangeal (MCP) joint flexed between 60° and 75°, a longitudinal incision of approximately 1 cm was made over the joint. The extensor tendon was split longitudinally to expose the base of the proximal phalanx. Once manual reduction was achieved, a 0.9 mm guidewire was inserted along the longitudinal axis of the phalanx in an antegrade manner under fluoroscopic guidance. The position of the guidewire was confirmed with fluoroscopy. Following this, a drill was used over the guidewire, and appropriate screw length was determined from preoperative imaging. A 2.2 mm or 3.0 mm SpeedTip cannulated compression screw (Medartis, Basel, Switzerland) was selected based on phalanx size, with 2.2 mm screws being used in most cases. The screw was advanced until fully buried below the cartilage line, with precaution taken to avoid over-insertion given the self-drilling, self-tapping nature of the implant. Furthermore, to prevent rotational deformity, the proximal phalanx was held firmly as the implant was inserted. Rotational alignment was verified clinically by confirming finger cascade after full screw insertion. Following confirmation, the skin was then closed with 2-0 nylon suture.

One point to be careful about when performing CCS fixation for transverse fractures is that this method only allows for forward movement, and backward movement is nearly impossible due to the nature of the screw. As an antegrade approach was utilized, greater caution is required in cases where the fracture line is more proximal to the base of the phalanx. To ensure the CCS does not pass the fracture line, repeated monitoring using fluoroscopy is required to confirm the position of the screw.

### 2.2. Postoperative Management, Rehabilitation, and Outcome Measurement

Postoperatively, patients were placed in a volar splint with the hand in an intrinsic-plus position. This was then transitioned to a custom forearm-based radial (second and third digit fractures) or ulnar gutter splint (fourth and fifth digit fractures) within five to seven days. Patients were then commenced on active and passive-assisted range of motion (ROM) exercises within one week of surgery, with progression to strengthening as tolerated during follow-up visits. The postoperative protocol was modified depending on the needs of the concomitant injuries.

Radiographic union and healing were evaluated on plain radiographs taken at every follow-up visit. Radiographic union was defined following the standards set by del Piñal [13] and Pun [24] where presence of trabecular bridging, bone consolidation, callus formation, and acceptable alignment were assessed on plain radiographs. Acceptable alignment for phalangeal fracture healing were defined as less than 10° angulation in the sagittal and/or coronal planes, where up to 20° angulation was accepted in the sagittal plane [24]. However, alongside radiographic union clinical assessment based on functional outcomes was accomplished to conclude complete fracture healing.

To measure functional outcome, Total Active Motion (TAM; active flexion of metacarpophalangeal, proximal interphalangeal, and distal interphalangeal joints minus the extension deficits in these joints) was measured using a handheld goniometer by an independent assessor (physician assistant) in every follow-up visit. Quick Disabilities of the Arm, Shoulder and Hand (QuickDASH) outcome scores was recorded to measure patient-reported outcomes. Finally, postoperative complications were defined as infection, loss of fixation, hardware failure, malrotation, nonunion, malunion, scar tenderness, metal allergy, delay in return to activities of daily living, or any need for repeat surgical intervention. 

## 3. Results

In this case series, 16 closed transverse and two short oblique fractures were treated in a total of 16 patients with a mean age of 51.1 years (SD 13.0, range 24–71). There were two patients sustaining two phalangeal fractures in their operating hand. All patients were right hand dominant with no significant comorbidities and all surgeries were performed within 10 days of injury. Further patient demographics and baseline characteristics are summarized in Table 1. Operative details entail three (18.8%) fractures that were fixed with a 3.0 mm headless cannulated screw, two of which involved the second digit and one involving the fourth. The remaining 15 (83.3%) fractures were fixed with a 2.2 mm headless screw. The mean screw length was 25.9 mm (SD 1.1, range 24–28) (Table 2).

**Figure 1:**
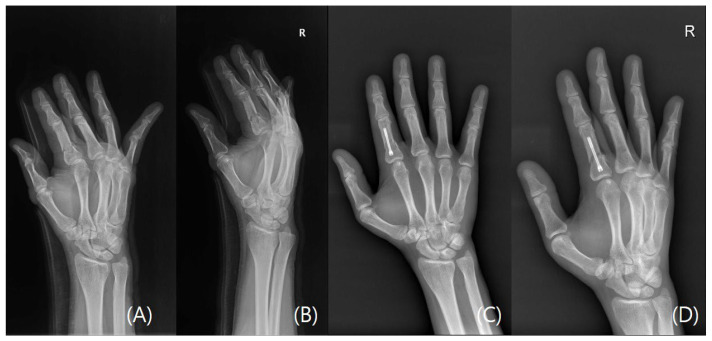
A case of a right second digit transverse proximal phalangeal fracture. (**A**) Preoperative plain radiograph Posteroanterior view. (**B**) Preoperative plain radiograph oblique view. (**C**) Postoperative plain radiograph Posteroanterior view taken at last follow-up visit. (**D**) Postoperative plain radiograph oblique view taken at last follow-up visit. The letter R refers to the right hand.

**Figure 2:**
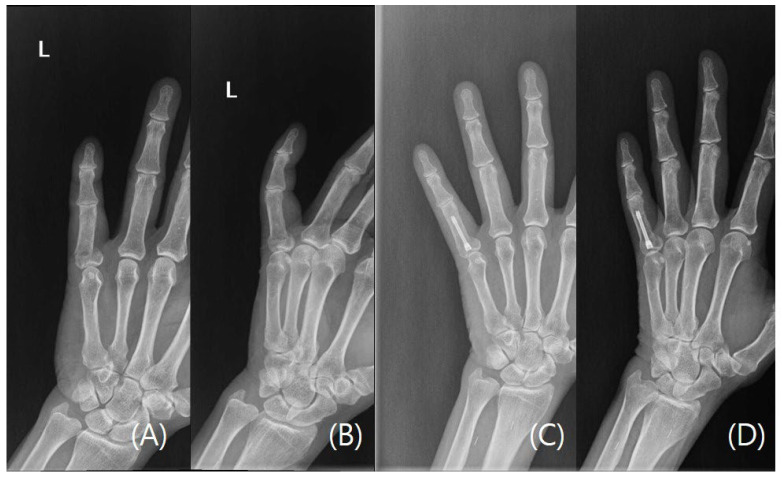
A case of a left fifth digit transverse proximal phalangeal fracture. (**A**) Preoperative plain radiograph Posteroanterior view. (**B**) Preoperative plain radiograph oblique view. (**C**) Postoperative plain radiograph Posteroanterior view taken at last follow-up visit. (**D**) Postoperative plain radiograph oblique view taken at last follow-up visit. The letter L refers to the left hand.

**Figure 3:**
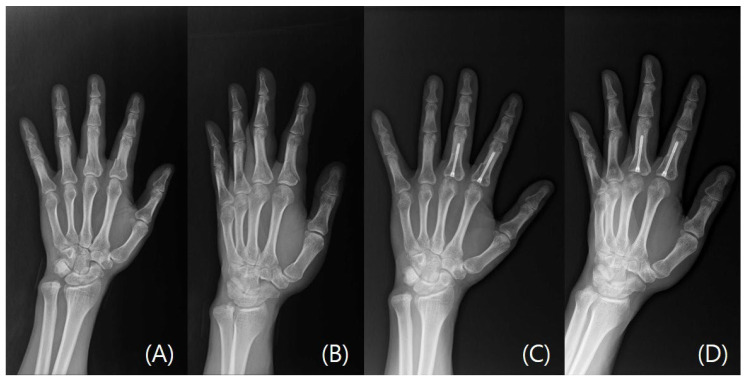
A case of left second and third digit short oblique proximal phalangeal fractures. (**A**) Preoperative plain radiograph Posteroanterior view. (**B**) Preoperative plain radiograph oblique view. (**C**) Postoperative plain radiograph Posteroanterior view taken at last follow-up visit. (**D**) Postoperative plain radiograph oblique view taken at last follow-up visit.

**Figure 4:**
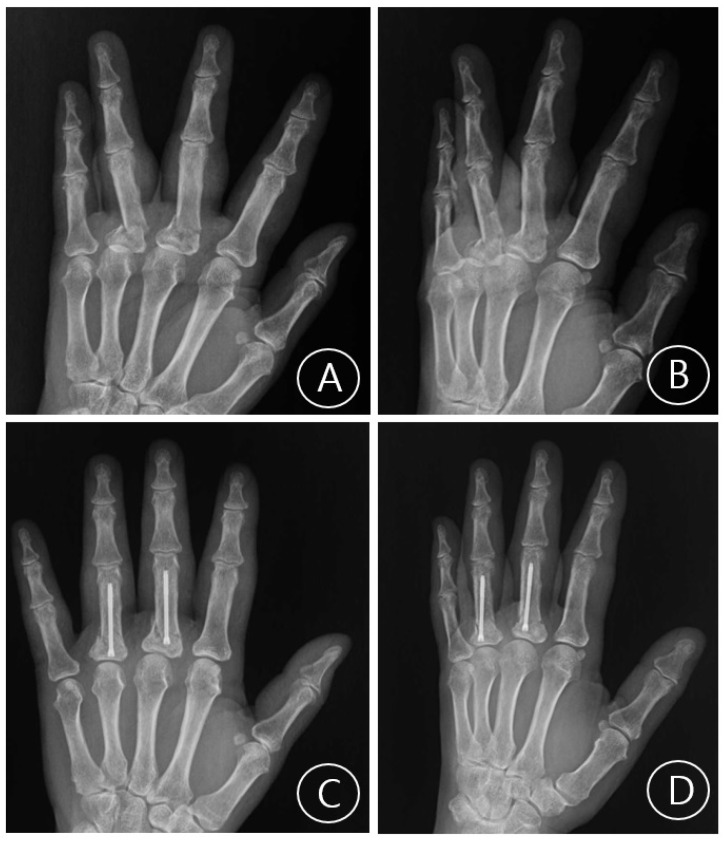
A case of a left third and fourth digit transverse proximal phalangeal fractures. (**A**) Preoperative plain radiograph Posteroanterior view. (**B**) Preoperative plain radiograph oblique view. (**C**) Postoperative plain radiograph Posteroanterior view taken at last follow-up visit. (**D**) Postoperative plain radiograph oblique view taken at last follow-up visit.

Mean postoperative follow-up was 11.0 weeks (SD 3.8, range 6–21) and all fractures were healed at the latest follow-up appointment verified with plain radiographs (examples cases, Figure 1, Figure 2, Figure 3 and Figure 4). No patient required reoperation for malrotation, malunion, delayed union, MCP joint stiffness, screw migration, or infection. Furthermore, no patients exhibited allergic or any adverse reaction to the implant and no postoperative complications were observed. Through patient-reported testimonies, all working patients resumed full duties without significant limitations and non-workers have returned to activities of daily living without any limitations. Mean TAM was 216.0° (SD 7.7°, range 200–230°) and mean QuickDASH was 10.1 (SD 3.8, range 5–16) (Table 3).

## 4. Discussion

This study demonstrates that antegrade CCS fixation is a feasible and safe procedure for unstable proximal phalanx fractures, resulting in consistent radiographic union, excellent function outcomes, and a low complication profile. The primary goal of surgical intervention for these common hand injuries is to provide a construct stable enough for early mobilization, thereby minimizing tendon adhesions and joint stiffness that frequently complicate treatment [25,26]. Our findings suggest this minimally invasive technique effective achieves this objective. However, given the small sample size for this case series and short-term follow-up period, these findings should be interpreted as preliminary.

The clinical outcomes observed in our case series are consistent with the growing body of literature supporting IMHS fixation. The mean TAM of 216.0° in our series is comparable to the pooled mean TAM of 237.0° reported in a recent systematic review of 204 proximal phalangeal fractures [27]. Similarly, our mean QuickDASH score of 10.1 aligns well with the averages of 3.6 and 6.2 reported in other series [25,27]. When compared directly to other fixation methods, intramedullary screws offer distinct advantages. A comparative study by Silins [14] found that patients treated with intramedullary screws had significantly better TAM, a shorter duration of work disability (5.6 vs. 9.9 weeks), and a much lower rate of hardware removal (17.6% vs. 93%) compared to those treated with plate fixation. These findings align well with known drawbacks of plate fixation, which has reported complication rates as high as 35% due to issues involving stiffness and the need for secondary hardware removal [28].

Biomechanical studies further support these clinical findings, demonstrating that intramedullary screws provide robust stability [6,29]. A study by Rausch [12] found that intramedullary compression screws offered the most stable constructs under bending forces when compared to plates and K-wires, with K-wires performing significantly worse across all testing modalities. For the short oblique fractures included in our case series, a cadaveric study by Miles [22] demonstrated that IMHCS fixation provides stability equivalent to that of plate fixation when subjected to simulated active ROM.

Beyond the choice of implant, specific technical considerations are critical to optimizing outcomes, particularly the insertion pathway. Our preference for an antegrade insertion pathway is deliberate, as it provides a direct entry point at the proximal phalanx base while avoiding disruption to the extensor tendon mechanism and PIP joint articular surfaces [2,4,30]. A recent biomechanical analysis by Gallardo-Calero [31] further supports this, concluding that antegrade fixation is more stable than retrograde fixation for these fractures. However, this technique requires precision. Over-advancement of the screw is a critical risk that can lead to joint penetration and mechanical impingement, making intraoperative fluoroscopic monitoring essential [4,32]. Similarly, applying appropriate torque is crucial, as over-tightening can cause fracture collapse and shortening rather than stable compression in cases with poor cortical support [28].

Furthermore, we favor an intra-articular entry point over a trans-articular one. A trans-articular approach, which would cross MCP joint from the metacarpal head, was intentionally avoided to prevent iatrogenic injury to the articular cartilage of the metacarpal head [4,30]. The intra-articular method confines the necessary cartilage defect to a small portion of the proximal phalanx base alone. While creating any articular defect is a concern, a cadaver study by Borbas [30] quantified this defect, finding that a 2.2 mm antegrade screw creates a defect of approximately 4.6% of the proximal phalanx’s articular surface, while a 3.0 mm screw affects 8.5%. While the long-term consequences of such defects in non-load-bearing upper extremity joints are not fully known, a multicenter case series on IMHS fixation of metacarpal fractures by Warrender [33] revealed headless screws to not leave large cartilage defects as all screw entry sites were filled with fibrocartilage and remain in congruence with the metacarpal head, inferring such technique to confer minimal to no significant sequelae. Importantly, our series lacked long-term MCP joint clinical follow-up, and whether the articular entry-point defect results in clinically relevant sequelae over time remains an important area for future investigation.

A key advantage of this technique is that it provides rigid internal fixation while minimizing violation of the extensor apparatus and surrounding soft tissues [6,28]. Unlike plate osteosynthesis, which requires extensive dissection that can lead to scarring and diminished motion, the percutaneous placement of an intramedullary screw, the very technique employed in the present study, preserves the delicate soft tissue envelope [2,4,34,35]. This stable fixation allows for the immediate initiation of postoperative ROM therapy, which is crucial for preventing stiffness and optimizing functional recovery. Furthermore, the headless design allows the screw to be completely buried beneath the articular surface, removing the need for routine hardware removal and avoiding interference with joint mechanics. Despite these benefits, potential drawbacks and complications must be considered. Besides the concern for iatrogenic damage to the articular cartilage mentioned above, other reported complications, though not observed in our series, include screw unraveling, particularly in younger patients with denser bones, and loss of fixation in long oblique fracture patterns [4,16]. As such, careful surgical technique, including pre-drilling in dense bone and frequent fluoroscopic monitoring, is recommended to mitigate these risks.

The success of CCS fixation is highly dependent on appropriate fracture selection. As reported by other studies this technique is best suited for transverse and short oblique extra-articular fractures of the proximal phalanx shaft or base, where there is adequate bone stock for screw purchase [13,36]. Conversely, its use is relatively contraindicated for long oblique fractures, where compression can lead to shortening and collapse, highly comminuted fractures, and marginal subchondral fractures, which may not provide sufficient purchase for screw threads and introduce the risk of splitting [4,36]. For such complex cases, the utilization of a more complex construct such as a “Y-strutting” or a dual-screw technique as described by del Piñal [13]—or interfragmentary screws as described by McQuillan [37]—is recommended. Absolute contraindications include fractures with an open epiphysis or active infection [2,13]. In our case series, it is important to note that there was only one case entailing two short oblique fractures, despite its favorable outcome the authors understand its limitation in further supporting the efficacy of intramedullary screw fixation for short oblique fracture cases.

This study has several noteworthy limitations, including its retrospective design, small sample size, and the absence of a direct comparison group. The retrospective nature introduces a risk of selection bias, and the small sample size limits the generalizability of our findings. Although our results are encouraging and align with the existing literature, the lack of a control group treated with an alternative fixation method prevents a definitive conclusion regarding the superiority of CCS fixation. Furthermore, the mean follow-up of 11.0 weeks, while sufficient for assessing fracture union and early functional recovery, is inadequate to evaluate long-term outcomes such as delayed stiffness, implant-related cartilage sequelae at the MCP joint entry point, or delayed hardware issues. Future studies with longer follow-up durations are needed to fully characterize the long-term safety profile of this technique.

## 5. Conclusions

In conclusion, antegrade fixation with cannulated compressive screws demonstrates feasibility, short-term safety, and excellent early functional outcomes in carefully selected transverse and short oblique proximal phalanx fractures, supporting its role as a viable, minimally invasive alternative. Given the retrospective design, small sample size, and limited follow-up duration of the present series, these findings should be interpreted as preliminary evidence rather than definitive proof of superiority over other fixation methods. Surgeons must remain mindful of the technique’s specific indications and potential complications. Further large-scale, prospective studies with longer follow-up are warranted to compare this method directly with other fixation modalities and evaluate long-term functional and articular outcomes.

## Figures and Tables

**Table 1:** Patient Demographics.

Demographics	Value
No. of Patients	16
Mean Age (years)	51.1 (13.0, 24–71) ^1^
Sex	
Male	11 (68.8%)
Female	5 (31.3)
Hand Involvement	
Right	5 (31.3%)
Left	11 (68.8%)
Digit Fractured	
1st	1 (5.6%)
2nd	6 (33.3%)
3rd	2 (11.1%)
4th	5 (27.8%)
5th	4 (22.2%)
Level of Fracture	
Base	14 (77.8%)
Shaft	4 (22.2%)

^1^ Mean (SD, range).

**Table 2:** Operative characteristics.

Digit	2.2 mm	3.0 mm	Total
1st	1 (6.7%)	0	1 (5.6%)
2nd	4 (26.7%)	2 (66.7%)	6 (33.3%)
3rd	2 (13.3%)	0	2 (11.1%)
4th	4 (26.7%)	1 (33.3%)	5 (27.8%)
5th	4 (26.7%)	0	4 (22.2%)
Total	15	3	18

**Table 3:** Postoperative outcomes.

Outcomes	Value
Follow-Up (weeks)	11.0 weeks (3.8, 6–21)
TAM	216.0° (7.7°, 200–230°)
QuickDASH	10.1 (3.8, 5–16)

Mean (SD, range).

## Data Availability

The data presented in this study are available on request from the corresponding author due to patient confidentiality and privacy restrictions.

## Abbreviations

The following abbreviations are used in this manuscript:

DIP	Distal Interphalangeal
PIP	Proximal Interphalangeal
CCS	Cannulated Compressive Screws
IMHS	Intermedullary Headless Screws
MCP	Metacarpophalangeal
TAM	Total Active Motion
ROM	Range of Motion
QuickDASH	Quick Disabilities of the Arm, Shoulder, and Hand
